# “When the going gets tough, who keeps going?” Depletion sensitivity moderates the ego-depletion effect

**DOI:** 10.3389/fpsyg.2014.00647

**Published:** 2014-06-24

**Authors:** Stefanie J. Salmon, Marieke A. Adriaanse, Emely De Vet, Bob M. Fennis, Denise T. D. De Ridder

**Affiliations:** ^1^Department of Marketing, University of GroningenGroningen, Netherlands; ^2^Clinical and Health Psychology Department, Utrecht UniversityUtrecht, Netherlands; ^3^Strategic Communication Chairgroup, Wageningen UniversityWageningen, Netherlands

**Keywords:** ego-depletion, depletion sensitivity, self-control, self-control endurance, individual differences

## Abstract

Self-control relies on a limited resource that can get depleted, a phenomenon that has been labeled ego-depletion. We argue that individuals may differ in their sensitivity to depleting tasks, and that consequently some people deplete their self-control resource at a faster rate than others. In three studies, we assessed individual differences in depletion sensitivity, and demonstrate that depletion sensitivity moderates ego-depletion effects. The Depletion Sensitivity Scale (DSS) was employed to assess depletion sensitivity. Study 1 employs the DSS to demonstrate that individual differences in sensitivity to ego-depletion exist. Study 2 shows moderate correlations of depletion sensitivity with related self-control concepts, indicating that these scales measure conceptually distinct constructs. Study 3 demonstrates that depletion sensitivity moderates the ego-depletion effect. Specifically, participants who are sensitive to depletion performed worse on a second self-control task, indicating a stronger ego-depletion effect, compared to participants less sensitive to depletion.

## INTRODUCTION

A wide variety of studies has demonstrated that the human capacity to exert self-control is limited (Baumeister et al., 1998, 2007). Whereas people are oftentimes well able to control their impulses, from time to time impulsive behavior aimed at short term gratification takes over and overrules behavior that is more beneficial in the long run. According to the limited strength model of self-control by Baumeister et al. (1998), self-control operates like a muscle that gets tired after repeated exertion: after an initial act of exerting self-control, like suppressing one’s urge to eat or act in an aggressive manner, individuals do not have sufficient self-control resources left to exert self-control in a second task, such as solving anagrams or endured performance on a hand grip task, an effect known as “ego-depletion” (Baumeister et al., 1998; Muraven et al., 1998; Schmeichel et al., 2003).

The ego-depletion effect has been demonstrated in many settings, using a great variety of tasks and measures (Hagger et al., 2010). Ego-depletion is now considered to be a robust phenomenon. It has also been demonstrated that self-control exertion is dependent on individual differences, such as experience with a certain ego-depleting task (Muraven et al., 1999), or motivation to perform well on this task (Muraven and Slessareva, 2003; Sato et al., 2010), which may compensate for the lack of self-control resulting from previous self-control exertion. Also lay theories about self-control have been found to predict levels of ego-depletion. When people hold the personal belief that self-control is a limited resource, they perform worse on a subsequent self-control task, than when they believe the self-control resource to be unlimited (Job et al., 2010). Notwithstanding the relevance of these concepts affecting self-control performance, we propose another, more generic concept that may affect self-control performance across a variety of ego-depleting situations, which we label “depletion sensitivity.” Specifically, we suggest that individuals may differ in the rate with which the self-control resource gets depleted as a consequence of depleting tasks and circumstances. We argue that some individuals will deplete their self-control resources faster than others when exerting self-control, and that sensitivity to depletion predicts ego-depletion distinct from other individual and situational differences that may influence self-control performance. In terms of the muscle metaphor, we propose that the “self-control muscle” of some people has more endurance than the muscle of others. For instance, some individuals may deplete their self-control resources only to a minor extent even after resisting numerous unhealthy food temptations during a party, whereas other people’s resources may already get depleted after having resisted one instance of buying unhealthy foods during a brief grocery shopping trip.

Taking the muscle metaphor of ego-depletion as a point of departure, our novel concept of depletion sensitivity bears two implications. First, people need muscle power to be able to exert effort at a certain moment. We posit that such power entails one’s situation-invariant level of trait self-control, or individuals’ more general capacity to exert self-control (Baumeister and Alquist, 2009). High levels of trait self-control are related to several positive long-term outcomes, such as more academic success and less binge eating (Tangney et al., 2004; De Ridder et al., 2012). Without a sufficient level of overall trait self-control, people will be less likely to exert self-control at a certain moment. Importantly, we state that besides muscle power, the endurance of the muscle is relevant as well in predicting the exertion of effort. At this point, depletion sensitivity comes into play. Whereas trait self-control may affect the extent to which people will exert self-control in the first place, depletion sensitivity taps into differences in the extent to which people are able to repeatedly keep exerting self-control over time. We argue that two individuals possessing similar levels of trait self-control may still differ in how fast their self-control resource gets depleted. We thus propose that depletion sensitivity represents muscle endurance, whereas trait self-control refers to the overall power of the muscle. Importantly, we do not expect these concepts to be unrelated. Individuals with more muscle power, who have a higher level of trait self-control, may be less sensitive to ego-depletion as they may have a larger self-control resource to draw from.

A second implication of our reasoning regarding depletion sensitivity bears that the effects of trait self-control and depletion sensitivity may affect subsequent self-control performance in different ways. In line with previous studies we expect that trait self-control has an overall effect on self-control behavior, regardless of whether or not people are in a state of ego-depletion, and does not necessarily moderate the effect of ego-depletion on self-control behaviors (Schmeichel and Zell, 2007). It should be noted though that whereas the majority of studies only revealed a main effect of trait self-control on the exertion of self-control, a limited number of studies did report a moderating effect of trait self-control on self-control performance (e.g., Muraven et al., 2005; DeWall et al., 2007; Gailliot et al., 2007; Imhoff et al., 2013). The results of these studies are, however, inconclusive. Whereas a small number of studies found a buffering effect of trait self-control, such that individuals high in trait self-control showed less self-control failure under conditions of ego-depletion, compared to individuals low in trait self-control (Muraven et al., 2005; DeWall et al., 2007; Gailliot et al., 2007), other studies failed to find such an effect (Gailliot and Baumeister, 2007; Stillman et al., 2009).

In view of these inconclusive findings, we propose that another individual difference may be important, which we label depletion sensitivity. Importantly, we argue that depletion sensitivity reflects the rate at which resources are drained *as a result of self-control demanding task requirements.* In line with this operationalization of depletion sensitivity, our expectations are thus slightly different than for trait self-control. As depletion sensitivity refers to how fast one’s self-control resource gets depleted, we expect individuals who are sensitive to depletion to be less able to exert self-control on a second self-control task compared to individuals who are less sensitive to depletion. In other words, we expect depletion sensitivity to influence the exertion of self-control under circumstances of ego-depletion. Therefore, we particularly expect an interaction effect between self-control task attributes and depletion sensitivity.

Surprisingly, the assumption that individuals may differ in depletion sensitivity has not been examined up to now. The primary aim of the present research is to demonstrate the relevance of the construct of depletion sensitivity. Specifically, in the present studies we investigate the proposition that individuals differ in their sensitivity to depleting tasks by examining the hypothesis that depletion sensitivity moderates the effect of ego-depletion on a subsequent self-control task.

Study 1 assesses whether individual differences in sensitivity to ego-depletion exist, by employing the Depletion Sensitivity Scale (DSS), a novel scale that was designed to measure the rate at which individuals’ self-control resources get depleted in response to self-control requiring conditions. Study 2 examines the pattern of correlations of depletion sensitivity with trait self-control (Tangney et al., 2004), state self-control (Ciarocco et al., 2010), lay beliefs about willpower (Job et al., 2010), impulsivity (Whiteside and Lynam, 2001; Cyders et al., 2007), and fatigue (Smets et al., 1995). Study 3 investigates whether depletion sensitivity moderates the effect of a depleting task on a subsequent self-control task.

## STUDY 1

The aim of the first study was to examine whether individual differences in ego-depletion exist.

### MATERIALS AND METHODS

#### Participants and procedure

Seventy-five participants (25.33% men) drawn from a community sample, with a mean age of 26.55 years (SD = 2.94) were recruited via social media, and voluntary participated in an online study. Participants completed the DSS and some demographic variables.

#### Depletion sensitivity scale

First, a pool of 30 items that were deemed relevant to the concept of depletion sensitivity was generated by the five authors. The items, using Likert statements, all specified a situation in which people’s self-control resources may become depleted, for instance after actively inhibiting impulses or after making a range of decisions (e.g., Baumeister et al., 1998; Vohs et al., 2008; Hagger et al., 2010), followed by a statement on the experience of depletion, an example item being: “After I have made a couple of difficult decisions, I will be mentally fatigued.” Based on group discussions, items that showed too much overlap with other items, or items that did not exclusively seem to measure depletion sensitivity, were excluded from the scale. From the initial pool of items, 15 items were selected to be included in the DSS (see **Table 1**). All items are rated on 7 point scales ranging from 1 (*totally disagree*) to 7 (*totally agree*). High scores on these items are expected to indicate high depletion sensitivity.

**Table 1:** Factor loadings of 15 items on the depletion sensitivity factor, Study 1.

Question	Factor loading
1. When I’m tired, I can’t say no	0.228
**2. After I have worked very hard at something, I am not good at reloading to start a new task**	0.526
**3. I get mentally fatigued easily**	0.714
**4. When I am (mentally) fatigued, I am easily tempted to do things that are actually no good for me**	0.645
**5. After I have made a couple of difficult decisions, I can be truly mentally “depleted”**	0.497
**6. After I exerted a lot of mental effort, I need to take a rest first before I can do another complicated task**	0.655
**7. It is hard for me to persist with a difficult task**	0.715
8. When I’m tired, I have difficulties doing something that needs to be done, instead of doing something fun (e.g., studying instead of watching TV)	0.297
9. I cannot make a good decision when I’m stressed	0.385
**10. When I’m tired, I have difficulties to suppress my emotions whenever that’s necessary (for example: not falling out with someone you’re angry with)**	0.477
**11. I have difficulties focusing my attention after I exerted a lot of mental effort**	0.708
**12. When I’m tired I have difficulties concentrating**	0.574
**13. At the end of a working day I often have difficulties staying focused**	0.628
**14. When I’m tired I sometimes have difficulties to remain friendly or polite**	0.521
15. When I’m tired I rather buy something that I like, even when it’s expensive	0.135

*Bold items are included in the final version of the Depletion Sensitivity Scale.
*

### RESULTS

#### Factor analysis

Exploratory factor analysis with varimax rotation of the 15 items yielded 5 factors with eigenvalues greater than one (4.42, 1.57, 1.40, 1.27, and 1.07, respectively). However, as the scree plot revealed that the first factor was clearly dominant, the analysis was re-run constraining the analysis to one forced factor. Eleven items loaded ≥0.40 on this factor (based on the criterion proposed by Floyd and Widaman, 1995; *R*^2^ = 29.46%; factor loadings are presented in the **Table 1**). The four items with loadings below 0.40 were removed from the scale. The 11-item scale had good reliability, with a Cronbach’s alpha of 0.83. The mean score on the DSS was 4.13 (SD = 0.87), ranging from 2.09 until 6.09, indicating that there is substantial variability in depletion sensitivity scores.

### DISCUSSION

We employed the DSS to assess individual differences in sensitivity to ego-depletion. Scores on this scale demonstrate that individual differences in sensitivity to ego-depletion exist.

## STUDY 2

The aim of this study was to examine how the construct of depletion sensitivity is associated with related constructs to assess its convergent and discriminant validity.

### MATERIALS AND METHODS

#### Participants and procedure

Two hundred forty six participants (57.3% men) drawn from the online participant pool Amazon’s Mechanical Turk, with a mean age of 34.08 years (SD = 10.75), participated in an online study for money. Participants completed the DSS, the Trait Self-Control Scale (Tangney et al., 2004), the State Self-Control scale (Ciarocco et al., 2010), the lay beliefs about willpower scale (Job et al., 2010), the UPPS + P Impulsive Behavior Scale (Whiteside and Lynam, 2001; Cyders et al., 2007), and the Multidimensional Fatigue Inventory (MFI; Smets et al., 1995).

#### Measures

All items were rated on a 7 point scale ranging from 1 (*totally disagree*) to 7 (*totally agree*).

***Depletion sensitivity.*** Depletion sensitivity was measured by the 11-item DSS, as developed in Study 1 (Cronbach’s alpha = 0.92).

***Trait self-control.*** The 13-item version of the Trait Self-Control Scale (Tangney et al., 2004) measures individual differences in self-control, an example item being “I am good at resisting temptation.” (Cronbach’s alpha = 0.87). An index was created by averaging the scores on the items.

***State self-control.*** The State Self-Control Scale (Ciarocco et al., 2010) measures state self-control, an example item being “I feel sharp and focused.” The scale consists of 25 items (Cronbach’s alpha = 0.95). An index was created by averaging the scores on the items.

***Lay beliefs about willpower.*** The lay beliefs about willpower scale (Job et al., 2010), has 12 items (Cronbach’s alpha = 0.82) and consists of two subscales. One subscale, measuring individual beliefs in the unlimited ability to exert strenuous mental activity, consists of six items (Cronbach’s alpha = 0.84), an example item being “After a strenuous mental activity, you feel energized for further challenging activities.” The other subscale measuring individual beliefs in the unlimited capacity to resist temptations also consists of six items (Cronbach’s alpha = 0.81), an example item being “Resisting temptations activates your willpower and you become even better able to face new upcoming temptations.” An index was created by averaging the scores on the items.

***Impulsivity.*** The UPPS + P Impulsive Behavior Scale (Whiteside and Lynam, 2001; Cyders et al., 2007) has 59 items (Cronbach’s alpha = 0.96) and consists of five subscales. The first subscale measures urgency (12 items, Cronbach’s alpha = 0.92), which refers to the tendency to experience strong impulses, frequently under conditions of negative affect, an example item being “When I am upset, I often act without thinking.” The second subscale measures premeditation (11 items, Cronbach’s alpha = 0.90), referring to the tendency to think and reflect on the consequences of an act before engaging in that act, an example item being “I usually think carefully before doing anything.” Subscale three measures perseverance (10 items, Cronbach’s alpha = 0.86), which refers to an individual’s ability to remain focused on a task that may be boring or difficult, an example item being “I generally like to see things through to the end.” Subscale four measures sensation seeking (12 items, Cronbach’s alpha = 0.92), referring to a tendency to enjoy and pursue activities that are exciting, and an openness to try new experiences that may or may not be dangerous, an example item being “I generally seek new and exciting experiences and sensations.” Finally, the fifth subscale (added by Cyders et al., 2007) measures positive urgency (14 items, Cronbach’s alpha = 0.97), referring to the tendency to experience strong impulses under conditions of positive affect, an example item being “I tend to lose control when I am in a great mood.” An index was created by averaging the scores on the items. To create this index we recoded the items of the premeditation and perseverance subscales, such that higher scores on these scales indicate lack of premeditation and perseverance, implying more impulsivity. However, for ease of interpretation, the original scores of these subscales are used in **Table 2**, in which higher scores on these scales indicate higher levels of premeditation and perseverance.

**Table 2:** Means, SD and correlations of the DSS with subscales of the UPPS +P Impulsive Behavior Scale, Study 2.

	1	2	3	4	5	6
Depletion sensitivity (1)	–					
Positive urgency (2)	0.53	–				
Negative urgency (3)	0.64	0.85	–			
Premeditation (4)	-0.13*	-0.45	-0.40	–		
Perseverance (5)	-0.41	-0.48	-0.52	0.52	–	
Sensation seeking (6)	0.21	0.51	0.42	-0.26	-0.09^ns^	–
*M*	3.84	3.23	3.46	5.16	5.20	4.02
SD	1.20	1.55	1.26	1.00	0.97	1.39

All correlations significant at *p *< 0.01, except *significant correlation at *p *< 0.05. *ns *= non-significant.

***Fatigue.*** The MFI (Smets et al., 1995) measures five dimensions of fatigue, which are general fatigue, physical fatigue, mental fatigue, reduced motivation, and reduced activity. For the present purpose, we used the composite score of fatigue. The scale consists of 20 items (Cronbach’s alpha = 0.91), an example item being “It takes a lot of effort to concentrate on things.” An index was created by averaging the scores on the items.

### RESULTS AND DISCUSSION

Depletion sensitivity is moderately related to trait and state self-control, lay beliefs about willpower, impulsivity and fatigue, indicating that these scales measure conceptually distinct constructs. Furthermore, depletion sensitivity is moderately related to positive and negative urgency and perseverance, indicating that these aspects of impulsive behavior are related to depletion sensitivity, but measure conceptually distinct constructs as well (see Tables 2 and 3 for means and correlations).

**Table 3:** Means, SD and correlations of the DSS with related scales, Study 2.

	1	2	3	4	5	6
Depletion sensitivity (1)	-					
Trait self-control (2)	-0.62	-				
State self-control (3)	-0.61	0.64	–			
Lay beliefs (4)	-0.63	0.51	0.40	–		
Impulsivity (5)	0.53	-0.71	-0.67	-0.36	–	
Fatigue (6)	0.61	-0.73	-0.76	-0.50	0.52	–
*M*	3.84	4.62	5.09	4.16	3.30	3.16
SD	1.20	1.06	1.13	0.88	0.97	1.02

*All correlations significant at *p *< 0.01.
*

As a check of convergent validity, we assessed whether the factor structure found in Study 1 replicates in the current sample. Exploratory factor analysis with varimax rotation of the 11 items yielded 1 factor with an eigenvalue greater than one (6.05). Forced factor analysis with one factor demonstrated that, again, all 11 items loaded ≥0.40 on this factor (*R*^2^ = 55.02%).

The results show satisfactory convergent and discriminant validity. More specifically, the factor analysis replicated our previous results, thus indicating a reliable scale producing a similar (unidimensional) factor structure. In addition, the moderate relationships to the constructs assessed in the present study show that depletion sensitivity is partly related to these constructs (as it should be), while not showing so much overlap that it taps into the same constructs. Rather, in line with the factor analysis, the DSS measures a clearly defined, undimensional construct, related to but still distinguished from, related constructs. This allows us to further investigate the hypothesis that depletion sensitivity may moderate the effects of ego-depletion on a subsequent self-control task.

## STUDY 3

Study 3 investigates the moderating effect of depletion sensitivity on actual ego-depletion effects, measured by scores on a cognitive performance task consisting of complex reasoning problems. Previous research has shown that performance on complex reasoning tasks requires self-control and is thus sensitive to ego-depletion (Schmeichel et al., 2003; Fennis et al., 2009). Depletion sensitivity is expected to moderate effects of ego-depletion on performance on this task. Specifically, as depletion sensitivity refers to one’s ability to keep exerting self-control over time, we hypothesize that participants who are highly sensitive to depletion will perform worse on this second self-control task, indicating a stronger ego-depletion effect, compared to participants who report low scores on depletion sensitivity.

### MATERIALS AND METHODS

#### Participants and procedure

One hundred and seven students participated in this study for money or course credit. Upon arrival at the laboratory, participants completed the DSS, and the self-control scales. Participants were then randomly assigned to the depletion or non-depletion condition, and performed the “E-crossing task” (see ego-depletion manipulation). Next, participants performed the cognitive performance task. Finally, participants provided demographic information, were thanked and debriefed.

One participant was excluded from the analyses because he did not complete the E-crossing task. Moreover, three participants who were outliers on the dependent variable as indicated by the standardized residuals calculated in the main regression analysis (see main analyses) were excluded from the analyses [standardized residuals > 2 (*N* = 2) and <-2 (*N *= 1); Anderson et al., 2009]. The final sample thus consisted of 103 participants (65% men) with a mean age of 19.77 years (SD = 1.86).

***Ego-depletion manipulation.*** The cover story for the self-control task told participants that the task was about written media. Participants were given an article that had been allegedly published in a popular magazine. In the non-depletion condition, participants had to cross out all the letters “e” in this text. Participants in the depletion condition had to cross out all the letters “e” in the first part of the text, and thereafter, in the second part of the text, only the letters “e” that applied to certain complex rules, such as: “the letters ‘e’ that are two spaces removed from a consonant” (conform the procedure of Baumeister et al., 1998). This more complex task has been found to deplete participants as they have to override their first impulse to cross out all the letters “e.”

***Cognitive performance.*** The cognitive performance test was presented as part of a yearly student contest. The participant who answered most questions correctly would win 20 Euro, increasing participants’ motivation to perform well (Baumeister and Vohs, 2007). The cognitive performance task consisted of 15 problems involving logical reasoning and word-relation problems (Schmeichel et al., 2003), an example item being: “medicine is to illness, as law is to..... A: anarchy; B: discipline; C: treason; D: etiquette,” (correct answer is “A”). Participants were not required to complete the whole task, and were told they could quit this task whenever they wanted. The cognitive performance task, with the ratio between number of completed items and correct items serving as the most prevalent measure, has been identified as a valid indicator of ego-depletion (Schmeichel et al., 2003; Fennis et al., 2009). However, since the variation in our data regarding the number of completed items was limited (78% completed 15 items and 10% completed 0 items), in the present study we only take into account the number of items answered correctly as dependent variable.

### MEASURES

The scales that were administered were the same as in Study 2. All items were rated on 7-point scales ranging from 1 (*totally disagree*) to 7 (*totally agree*). Cronbach’s alpha’s for depletion sensitivity (0.84), trait self-control (0.83), and state self-control (0.93) were all satisfactory.

### RESULTS

#### Descriptives and randomization check

Mean scores and correlations are reported in **Table 4**. An ANOVA with ego-depletion condition as independent variable revealed no differences between conditions in depletion sensitivity (*F*(1,101) = 1.9; *p *= 0.17), trait self-control (*F* < 1), and state self-control (*F* < 1), indicating successful randomization.

**Table 4:** Means, SD and correlations Study 3.

	1	2	3	4
Depletion sensitivity (1)	–			
Trait self-control (2)	-0.43^**^	–		
State self-control (3)	-0.45^**^	0.30^**^	–	
Cognitive performance (4)	0.05	-0.05	-0.02	-
*M*	4.17	4.12	5.17	5.89
SD	0.87	0.81	0.77	3.24

** Significant difference at *p*< 0.01.

#### Main analyses

To determine whether there was an interaction effect between depletion sensitivity and ego-depletion condition on cognitive task performance, a regression analysis consisting of three steps was conducted. Ego-depletion condition was entered into the model in the first step. In step 2, state self-control, trait self-control and depletion sensitivity were entered. In the final step, the interaction variable between depletion sensitivity and condition was entered. All continuous independent variables were mean centered before being entered in the regression analysis (Aiken and West, 1991). See **Table 5** for the results of this regression analysis.

**Table 5:** Regression analysis of cognitive task performance, Study 3.

	β	*t*	*p*
**Step 1: *R*^2^ = 0.04, *F*(1,101) = 3.75, *p *= 0.056**
Depletion condition	-0.19	-1.94	0.056
**Step 2: *R*^2^ = 0.04, *F *< 1**
Depletion condition (0 = non-depletion, 1 = depletion)	-0.19	-1.91	0.06
State self-control	0.00	0.03	0.98
Trait self-control	-0.06	0.51	0.61
Depletion sensitivity	-0.01	-0.04	0.97
**Step 3: *R*^2^ = 0.12, *F*(5,97) = 2.74, *p *= 0.023**
Depletion condition × depletion sensitivity	-0.46	-3.07	<0.01
Depletion condition (0 = non-depletion, 1 = depletion)	-0.18	-1.90	0.06
State self-control	-0.01	-0.11	0.91
Trait self-control	-0.04	-0.33	0.74
Depletion sensitivity	0.35	2.15	0.03

**FIGURE 1:**
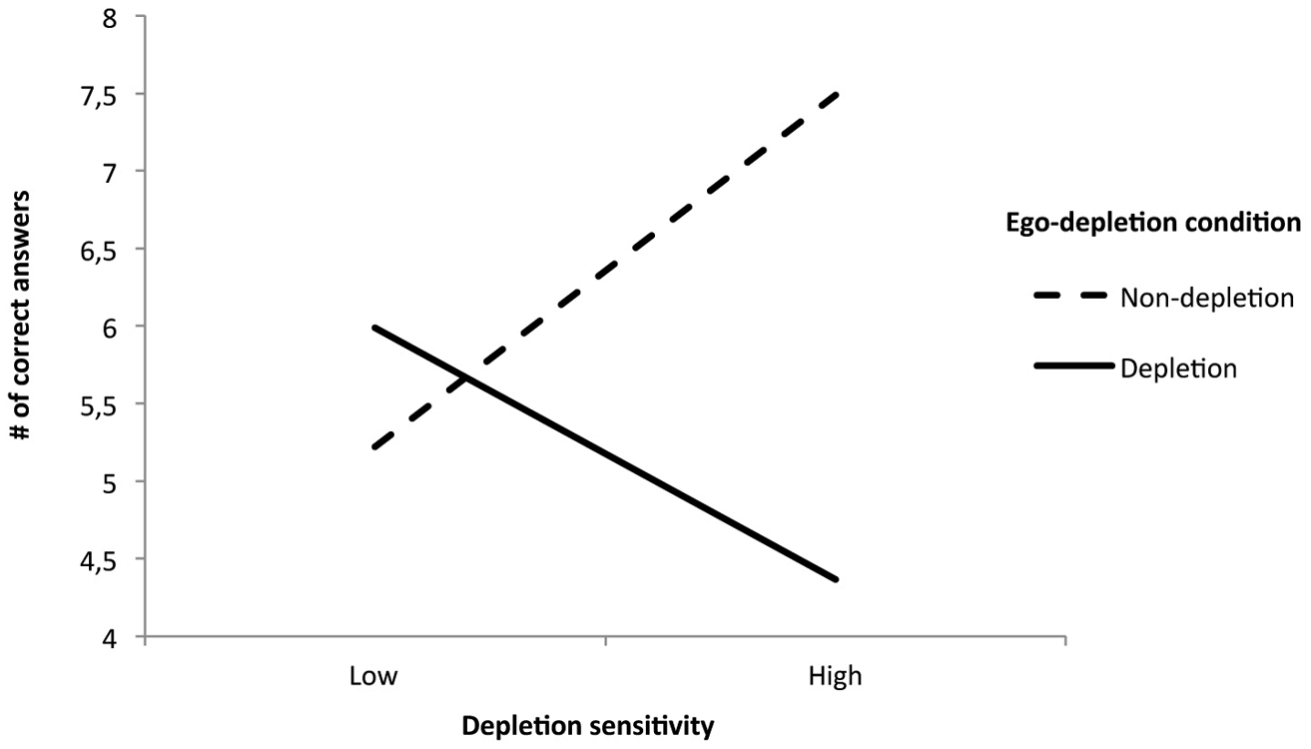
**The interaction between depletion sensitivity and ego-depletion condition on cognitive task performance, Study 3**.

The first step revealed a marginally significant effect (*p *= 0.056) of condition on task performance. Participants in the depletion condition had marginally less correct answers on the cognitive performance task (*M* = 5.29, SD = 3.53), compared to participants in the non-depletion condition (*M *= 6.51, SD = 2.83). Step 2 showed no significant effects of the control variables, and no main effect of depletion sensitivity (*p*’s > 0.61). The expected interaction between condition and depletion sensitivity on the number of correct answers was significant, β = -1.00, *t*(97) = -3.07,*p* = 0.003^1^ (see **Figure 1** for the plotted interaction). Simple slopes analyses showed that for participants high in depletion sensitivity (+1 SD; Aiken and West, 1991), there was a significant effect of ego-depletion condition on number of correct answers, β = -0.49, *t*(97) = -3.57,*p* = 0.001, whereas there was no effect of ego-depletion condition on number of correct answers for participants low in depletion sensitivity (-1 SD; Aiken and West, 1991), *p* = 0.40.

In order to test whether trait self-control has a similar moderating effect as depletion sensitivity, a second regression analysis was conducted to test the effect of ego-depletion condition, trait self-control and their interaction on cognitive task performance. Step 1 and 2 were similar to the first regression, but in the third step the interaction variable between ego-depletion condition and trait self-control was entered instead. Except for the first step (*p* = 0.056), none of the other steps were significant, *p*’s > 0.23.

### DISCUSSION

Results from Study 3 confirm our hypothesis that depletion sensitivity moderates the effect of ego-depletion on a subsequent self-control task. As expected, individuals who scored high on depletion sensitivity were more affected by the self-control task, in the sense that the ego-depletion effect was stronger for individuals high, than for individuals low in depletion sensitivity. Importantly, there was no interaction of trait self-control and ego-depletion condition, emphasizing the relative contribution of depletion sensitivity as compared to trait self-control.

## GENERAL DISCUSSION

The present studies show that individuals differ in their sensitivity to depleting circumstances (Study 1), and that this depletion sensitivity construct is conceptually distinct from related constructs such as trait self-control, state self-control, and lay beliefs about self-control (Study 2). Depletion sensitivity predicted self-control exertion under ego-depleting conditions (Study 3). The ego-depletion effect was stronger for individuals high, compared to individuals low in depletion sensitivity, as was shown by self-control performance on a cognitive performance task.

With the present research we aimed to explore deeper the conditions under which ego-depletion manifests itself. We demonstrated that some individuals are more sensitive to depletion inducing tasks than others, and will consequently deplete their self-control resources at a faster rate in the face of depleting circumstances. Our findings suggest that ego-depletion is not an all or nothing phenomenon that is equally likely to lead to self-control failure for all individuals, but rather that it is more flexible and depends on individual capacities to deal with potentially depleting circumstances. This finding is also in line with the more general finding that motivation (Muraven and Slessareva, 2003), perceived task difficulty (vanDellen et al., 2012), or personal beliefs about self-control (Job et al., 2010) may moderate ego-depletion effects (for an overview see Inzlicht and Schmeichel, 2012).

The present findings also point to new avenues for countering the adverse effects that are often associated with depletion, such as disadvantageous decision making, and poorer cognitive performance (Baumeister et al., 1998; Wang et al., 2010). Our findings suggest that it might be useful to investigate ways to decrease people’s sensitivity to depleting circumstances, and enhance their “muscle endurance.” Relatedly, future studies should investigate how people with high depletion sensitivity can protect themselves against depleting circumstances, for example by investigating the attentional and motivational processes in individuals with high and low depletion sensitivity (cf. Inzlicht and Schmeichel, 2012). Insight into these mechanisms may provide valuable information to teach depletion sensitive individuals how to deal with ego-depleting conditions.

It should be noted that in contrast with the small number of studies showing that trait self-control moderates the effect of ego-depletion on self-control behaviors (Muraven et al., 2005; DeWall et al., 2007; Gailliot et al., 2007; Imhoff et al., 2013), trait self-control was, unlike depletion sensitivity, not a significant moderator in the present studies. This underscores the relative contribution of the depletion sensitivity construct, as depletion sensitivity specifically captures the degree to which individuals are affected by depleting circumstances. Importantly, trait self-control and depletion sensitivity should not be viewed as completely unrelated as the correlations between these constructs were moderate (-0.62 and -0.43 in Studies 2 and 3). This finding indicates that, overall, people with more capacity to exert self-control are also less sensitive to depleting circumstances.

Our research is not without limitations. First, the third study involved participants from a student sample who were relatively young and well educated. In order to increase the external validity of our results, future research should examine the extent to which the findings of this study hold in a community sample. Furthermore, in the present studies, we only used cognitive tasks as manipulations and measures of ego-depletion. Moreover, there was no significant main effect of ego-depletion condition on task performance in Study 3, which is in contrast to previously found ego-depleting effects of the e-erasing task on secondary self-control tasks (e.g., Baumeister et al., 1998; Tice et al., 2007). Future studies on depletion sensitivity should include other (non-cognitive) types of manipulations and measures of ego-depletion, such as the handgrip task or an emotion-regulation task (Muraven et al., 1998).

Altogether, depletion sensitivity is a relevant concept in studying self-control processes. By identifying the mechanisms that determine who keeps going when the going gets tough and who does not we made a first crucial step in providing more detailed insight in self-control performance as affected by depletion sensitivity.

## Conflict of Interest Statement

The authors declare that the research was conducted in the absence of any commercial or financial relationships that could be construed as a potential conflict of interest.

## Footnotes

1 To control for a possible effect of three outliers on our results, we ran the same regression analysis including these outliers (with standardized residuals >2 [*N* = 2] and <-2 [*N *= 1]). The regression analysis shows that the interaction between ego-depletion condition and depletion sensitivity remains significant, β = -0.34, *t*(100) = -2.24, *p *= 0.027. There were no main effects of ego-depletion (*p *= 0.11), state self-control (*p* = 0.96), trait self-control (*p *= 0.84) and depletion sensitivity (*p = *0.12) on task performance. Simple slopes analyses showed that for participants high in depletion sensitivity (+1 SD; Aiken and West, 1991), there was a significant effect of ego-depletion condition on number of correct answers, β = -0.38, *t*(100) = -2.67, *p* = 0.007, whereas there was no effect of ego-depletion condition on number of correct answers for participants low in depletion sensitivity (-1 SD; Aiken and West, 1991), *p* = 0.64.
